# Engineering of Crystal and Domain Structures in Epitaxial Y:HfO_2_ Thin Films by YSZ Substrate Miscut

**DOI:** 10.1002/advs.202524377

**Published:** 2026-04-16

**Authors:** Jun Young Lee, Hyung‐Jin Choi, Kun Hee Ye, Haneul Choi, Byeong‐hyeon Lee, Min‐Seok Kim, Dong‐Hun Han, June Hyuk Lee, Sung Ok Won, Tae Heon Kim, Hye Jung Chang, Jung‐Hae Choi, Seung‐Hyub Baek

**Affiliations:** ^1^ Electronic & Hybrid Materials Research Center Korea Institute of Science and Technology Seoul Republic of Korea; ^2^ Center for Hydrogen Energy Materials Korea Institute of Science and Technology Seoul Republic of Korea; ^3^ Advanced Analysis and Data Center Korea Institute of Science and Technology Seoul Republic of Korea; ^4^ Department of Materials Science and Engineering Research Institute of Advanced Materials Seoul National University Seoul Republic of Korea; ^5^ Neutron Science Division Korea Atomic Energy Research Institute Daejeon Republic of Korea; ^6^ Division of Nanoscience and Technology KIST School Korea National University of Science and Technology Seoul Republic of Korea

**Keywords:** crystal structure, domain structure, epitaxial HfO_2_ thin films, ferroelectric, miscut substrate

## Abstract

The crystal and domain structures of epitaxial polymorphic oxide thin films are governed not only by intrinsic bulk energies but also by extrinsic factors arising from interactions with the underlying oxide substrates. Here, we investigate the effects of substrate miscut on the phase stability of epitaxial HfO_2_ films on yttria‐stabilized zirconia (Y:ZrO_2_, YSZ), employing a combined experimental and computational approach to map the thickness‐temperature phase diagram. High‐quality epitaxial 6% Y‐doped HfO_2_ films with atomically smooth surfaces were fabricated by magnetron sputtering on both nominally‐flat and 8°‐miscut (001) YSZ single‐crystal substrates. On nominally‐flat substrates, (100)‐oriented orthorhombic films form a two‐variant domain structure and evolve into (001)‐oriented monoclinic phases at larger thicknesses. On 8°‐miscut substrates, in contrast, a (100)‐oriented orthorhombic phase with a preferential population among the two variants is stabilized at small thicknesses, whereas a reoriented, (100)‐oriented monoclinic phase is favored at larger thicknesses, accompanied by a pronounced narrowing of the orthorhombic stability window. This work provides fundamental insight into the control of phase stability and domain architecture in polymorphic oxides, relevant to the integration of complex oxides in advanced electronic platforms.

## Graphical abstract

We investigate how YSZ substrate miscut influences crystal structure and domain formation in epitaxial Y‐doped HfO_2_ thin films. Using magnetron sputtering, high‐resolution X‐ray diffraction, atomic‐resolution scanning transmission electron microscopy, and first‐principles calculations, we systematically examine the characteristics of thickness‐ and temperature‐dependent phase transitions on nominally‐flat and 8°‐miscut substrates and constructed comparative phase‐diagram frameworks.

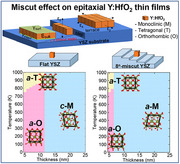

## 1 Introduction

Controlling the crystal and domain structures of functional oxide thin films is essential for both practical device engineering and the broader scientific understanding of functional oxides. The ability to manipulate crystal structure offers a powerful route for understanding how polymorphic phases compete and stabilize in oxide systems with complex free‐energy landscapes [1, 2, 3, 4, 5]. Functional oxide materials often host multiple metastable phases that lie close in energy, and subtle variations in strain state can shift the energy hierarchy, selectively stabilizing phases that are absent in the bulk [6, 7, 8, 9]. This sensitivity offers a unique opportunity to access otherwise hidden structural states and to investigate the emergent phenomenon.

One of the most prominent examples is the ferroelectric hafnium oxide (HfO_2_) thin films, which have rapidly emerged as a promising materials platform for next‐generation nanoelectronic devices—particularly for logic‐in‐memory architectures—owing to their CMOS compatibility, scalability down to a few nanometers, and the unexpected discovery of robust ferroelectricity in doped HfO_2_ systems [10, 11, 12, 13, 14, 15, 16]. HfO_2_, conventionally a non‐ferroelectric high‐*k* dielectric, can stabilize a polar orthorhombic phase exhibiting ferroelectricity when fabricated as nanoscale thin films subject to appropriate conditions. Typical dopants such as Si, Zr, Al, Y, Gd, and La aid in stabilizing the polar phase, yet their effectiveness depends critically on extrinsic factors—film thickness, annealing conditions, and electrode selection [17, 18, 19, 20].

Domain structures in ferroelectrics govern a spectrum of key performance parameters for devices, including leakage current, remanent polarization, coercive field, dielectric loss, retention, and fatigue endurance [21, 22]. These properties are dictated primarily by the polarization‐switching pathway and its interaction with defects, both of which are determined by the relative orientation between the allowed polarization axes of the crystal and the direction of the applied electric field. In addition, the structural and electronic character of the domain boundaries—whose symmetry, orientation, and charge state can differ markedly from those of the surrounding domains—further influences the electrical behavior of ferroelectric thin films.

Notably, the use of miscut substrates—characterized by step‐and‐terrace surface morphologies that break in‐plane symmetry—has emerged as a particularly effective method for domain engineering. In BiFeO_3_ thin films, four‐domain variants commonly form on nominally‐flat (*001*) SrTiO_3_, while introducing a 4° miscut toward the [100] or [110] direction reduces the domain multiplicity to two and one, respectively [23, 24]. These reductions not only simplify the domain landscape but also improve ferroelectric properties: single‐domain BiFeO_3_ films exhibit lower leakage current, reduced coercive field, and higher remanent polarization [18, 25]. Similarly, in Bi_2_Te_3_, a topological insulator, miscut‐induced step edges align the in‐plane orientation of van der Waals layers, suppressing twin domains and tuning electronic transport behavior [26, 27]. These findings highlight the crucial role of miscut direction and angle in guiding domain alignment, reducing structural complexity, and tailoring functional properties in epitaxial thin films.

While the effects of substrate miscut have been widely studied in oxide ferroelectrics, these prior works typically focus on systems with essentially fixed crystal structures, in which the miscut influences only the configuration of domain variants. In contrast, hafnia‐based ferroelectrics are highly polymorphic—especially with the metastable orthorhombic phase of interest—because multiple phases lie close in energy, making the crystal structure itself sensitive to extrinsic boundary conditions. As a result, a substrate miscut could modify not only domain selection but also the stabilization of the competing phases. Despite this unique opportunity for phase control, the influence of substrate miscut on orthorhombic hafnia‐based ferroelectrics remains unexplored.

In this work, we extend the framework of miscut‐enabled domain engineering to epitaxial HfO_2_ thin films, where phase and domain structures can be unambiguously identified [28, 29, 30, 31]. We present a comprehensive investigation of domain structure and phase stability in epitaxial 6 at.% Y‐doped HfO_2_ (YHO) thin films grown on nominally‐flat and intentionally 8°‐miscut (*001*) yttria‐stabilized zirconia (YSZ) single‐crystal substrates. Combining high‐resolution X‐ray diffraction (HRXRD), scanning transmission electron microscopy (STEM), and first‐principles thermodynamic modeling, we reveal a clear dependence of the stabilized crystal phase—monoclinic, orthorhombic, or tetragonal—on both film thickness and substrate geometry. The experimental results are strongly consistent with the phase diagram built by the first‐principle calculations. Our results show that substrate miscut modifies not only domain structures but also crystal phase stability, establishing miscut engineering as a versatile and previously underexplored strategy for tailoring the properties of HfO_2_‐based thin films.

## 2 Results and Discussion

### 2.1 Epitaxial YHO Thin Films on YSZ

**FIGURE 1:**
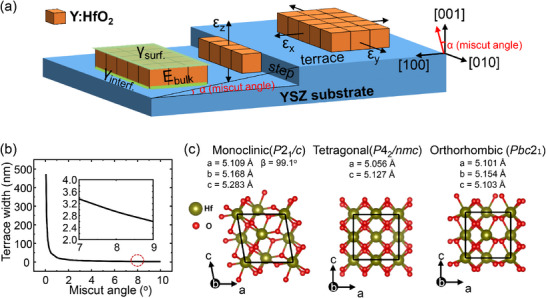
(a) Schematic illustration of the epitaxial growth of YHO thin films on a miscut YSZ substrate. (b) Terrace width on the surface of miscut YSZ substrates as a function of miscut angle. (c) Schematics of the HfO_2_ polymorphic unit cells and the axis conventions used in this work.

Figure 1a schematically illustrates the surface morphology of YSZ miscut substrates during the epitaxial growth of YHO films. The miscut surface is characterized by a regular array of atomic terraces separated by unit‐cell‐height steps, created by intentionally tilting the substrate surface by a miscut angle (α) from the exact (*001*) plane. On the *terrace regions*, the crystal structure of epitaxial YHO film is mainly governed by biaxial in‐plane (x–y) strain generated by the lattice mismatch between YHO and YSZ. This in‐plane strain induces lattice distortion, thereby stabilizing or suppressing specific polymorphic phases depending on the degree of mismatch and film thickness. Notably, anisotropic biaxial strain arises in epitaxial YHO films grown on (*001*) cubic YSZ substrates due to their intrinsic symmetry mismatch, as YHO can crystallize in monoclinic, orthorhombic, or tetragonal phases with unequal in‐plane lattice parameters, whereas YSZ maintains a cubic lattice with identical in‐plane parameters. Consequently, the biaxial epitaxial strain plays a crucial role in determining the lattice distortion and relative phase stability depending on the orientation and phase state of the YHO films. On the *step regions*, the local environment differs markedly from that of the atomically flat terraces. At the step edges, vertical lattice mismatch generates additional out‐of‐plane (z) strain components that locally perturb the otherwise biaxial epitaxial strain state. This local and pseudo‐periodic constraint may partially alter the stabilized energy state dictated by the biaxial strain on terraces, thereby modifying the ground‐state configuration. In addition, the interfacial energy between YHO and YSZ, together with the surface energy of the YHO film, further affects the stability of a particular crystal structure.

The miscut angle and direction must be carefully selected in order to control the density and orientation of surface steps that are sufficiently prominent to influence the overlying epitaxial HfO_2_ thin films. The relationship between the terrace width of the YSZ substrate and the miscut angle is shown in Figure 1b. As the miscut angle increases, the terrace width decreases, leading to a higher density of surface steps. The selection of an appropriate miscut angle depends critically on the growth mode of the thin film. In the step‐flow growth regime [32], where adatoms attach laterally to step edges without nucleating 2D islands on terraces, a relatively low miscut angle can selectively stabilize specific domain variants while suppressing the formation of competing ones. For instance, single‐domain SrRuO_3_ thin films can be achieved on SrTiO_3_ substrates with a miscut angle of approximately 1.9° [33]. In the layer‐by‐layer growth regime, in contrast, the miscut angle must be sufficiently large to suppress two‐dimensional nucleation on terraces, which would otherwise proceed in a manner similar to that observed on nominally‐flat substrates. Notably, YHO films grown on 4°‐miscut YSZ substrates exhibit intermediate behavior, in which neither the phase configuration characteristic of nominally‐flat substrates nor that stabilized on 8°‐miscut substrates is exclusively favored; instead, both phases are stabilized concurrently, reflecting a transitional regime between the two miscut extremes (see Figure S1). With respect to the choice of miscut direction, the [100] orientation was selected to minimize structural ambiguity at the step edges and to ensure consistent step orientation and density across the substrate surface.

Figure 1c schematically illustrates the crystal structures of the three primary phases—monoclinic (P2_1_/c), orthorhombic (Pbc2_1_), and tetragonal (P4_2_/nmc)—with their crystallographic axes. This is intended to provide a unified and consistent reference for the definition of the *a*, *b*, and *c* axes in each phase, which have often been inconsistently labeled or interpreted in prior literature. Throughout this paper, we adopt the axis conventions shown in Figure 1c [34]. Clear identification of these axes is critical not only for structural characterization but also for accurately describing domain orientation and epitaxial relationships. The monoclinic phase is the thermodynamically stable equilibrium phase of undoped HfO_2_ under ambient conditions. In the orthorhombic phase, which emerges as a metastable structure in doped HfO_2_ systems and is responsible for the onset of ferroelectricity, the crystallographic axes are orthogonal but non‐equivalent. This anisotropy gives rise to a spontaneous polarization oriented along the *c*‐axis, which defines the ferroelectric axis of the structure. In the tetragonal phase—which often emerges at high temperatures—the crystal structure features an elongated *c*‐axis with respect to the equivalent *a*‐ and *b*‐axes.

Yttrium (Y) was selected as a dopant owing to its narrow compositional window for stabilizing the metastable orthorhombic phase, as previously reported in ALD‐grown polycrystalline HfO_2_ thin films [35]. Notably, even a 1 at.% variation in Y concentration markedly alters the resulting phase: 33 nm‐thick epitaxial HfO_2_ films doped with 5, 6, and 7 at.% Y, grown on (*001*) YSZ under identical conditions, exhibit monoclinic, orthorhombic, and tetragonal structures, respectively (Figure S2). This pronounced compositional sensitivity provides an excellent platform for elucidating the effects of extrinsic parameters—such as substrate miscut and film thickness—on polymorphic phase formation. As the orthorhombic phase is the target functional phase, a Y concentration of 6 at.% was employed in subsequent experiments.

### 2.2 Thickness‐Dependent Phase Evolution of YHO Thin Films on Nominally‐Flat YSZ

**FIGURE 2:**
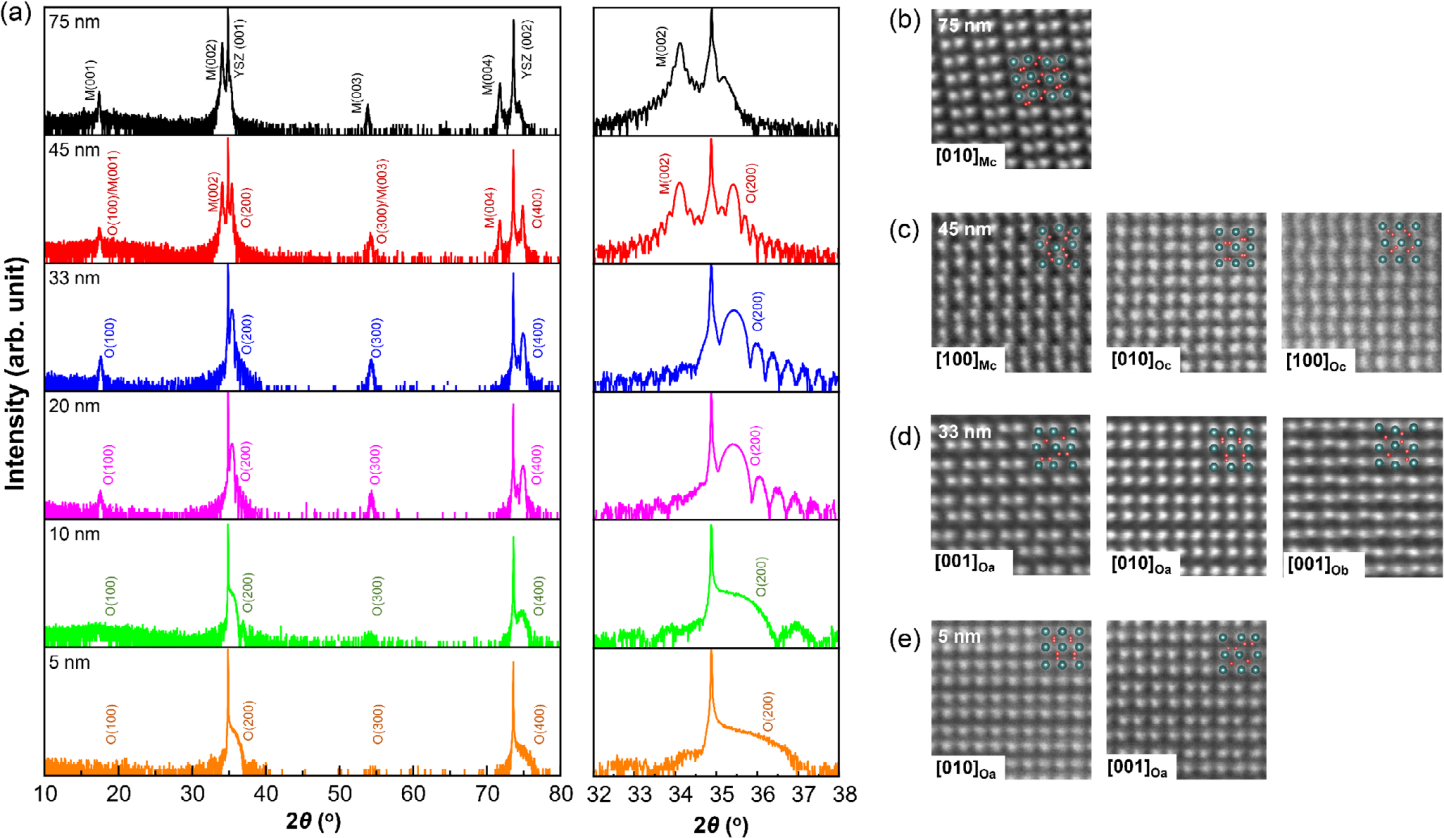
(a) HRXRD *θ*–2*θ* scans of epitaxial YHO thin films with various thicknesses grown on nominally‐flat YSZ substrates. The right panel shows a magnified view around the (001) YSZ peak. (b–e) Cross‐sectional atomic‐resolution STEM images, processed by inverse fast Fourier transform (FFT) filtering, of epitaxial YHO thin films with thicknesses of (b) 75 nm, (c) 45 nm, (d) 33 nm, and (e) 5 nm. Hf and O atoms are marked as green and red circles, respectively.

Figure 2a shows the HRXRD *θ*–2*θ* patterns of epitaxial YHO films with varying thicknesses grown on nominally‐flat (*001*) YSZ substrates. The second column of Figure 2a shows magnified views of the diffraction regions around the (002) reflection of the YSZ substrate. Clear satellite peaks corresponding to thickness fringes of the YHO films are observed around the main diffraction peaks, indicating atomically smooth film surfaces (Figure S3). In contrast to polycrystalline HfO_2_ thin films, the limited diffraction peaks available in epitaxial films render the XRD peak positions alone insufficient to precisely distinguish among the monoclinic, orthorhombic, and tetragonal phases and their orientations. In the ultrathin regime (5 and 10 nm), the XRD peaks are broadened, making it difficult to unambiguously determine the crystalline phase and its orientation solely from diffraction data. This issue becomes further complicated when possible epitaxial strain is taken into account, as such strain may shift the diffraction peak positions and hinder unambiguous phase identification. Therefore, high‐resolution TEM analysis is essential to resolve the atomic arrangements and zone‐axis orientations, providing complementary evidence to confirm the structural assignment of the ultrathin films.

Figures 2b–e provide high‐resolution high‐angle annular dark‐field scanning transmission electron microscopy (HAADF‐STEM) images that directly visualize the atomic arrangements, enabling identification of the crystal structures in real space. The film thickness is labeled in white text at the top‐left corner of each image, while the zone axis is denoted by black text within a white box (e.g., “[010]Mc” denotes the monoclinic phase (M) viewed along the [010] zone axis, with the *c*‐axis oriented along the out‐of‐plane direction). The combined XRD and TEM analyses confirm the phase identification: films ranging from 5 to 33 nm exhibit an orthorhombic phase, the 45 nm film contains a mixture of orthorhombic and monoclinic phases, and the 75 nm film is predominantly monoclinic. These results demonstrate that YHO films grown on nominally‐flat YSZ substrates undergo a pronounced thickness‐driven polymorphic evolution, transitioning from orthorhombic at small thickness to monoclinic at larger thicknesses.

### 2.3 Miscut Effect on the Domain Structure of Thick Monoclinic YHO Thin Films

**FIGURE 3:**
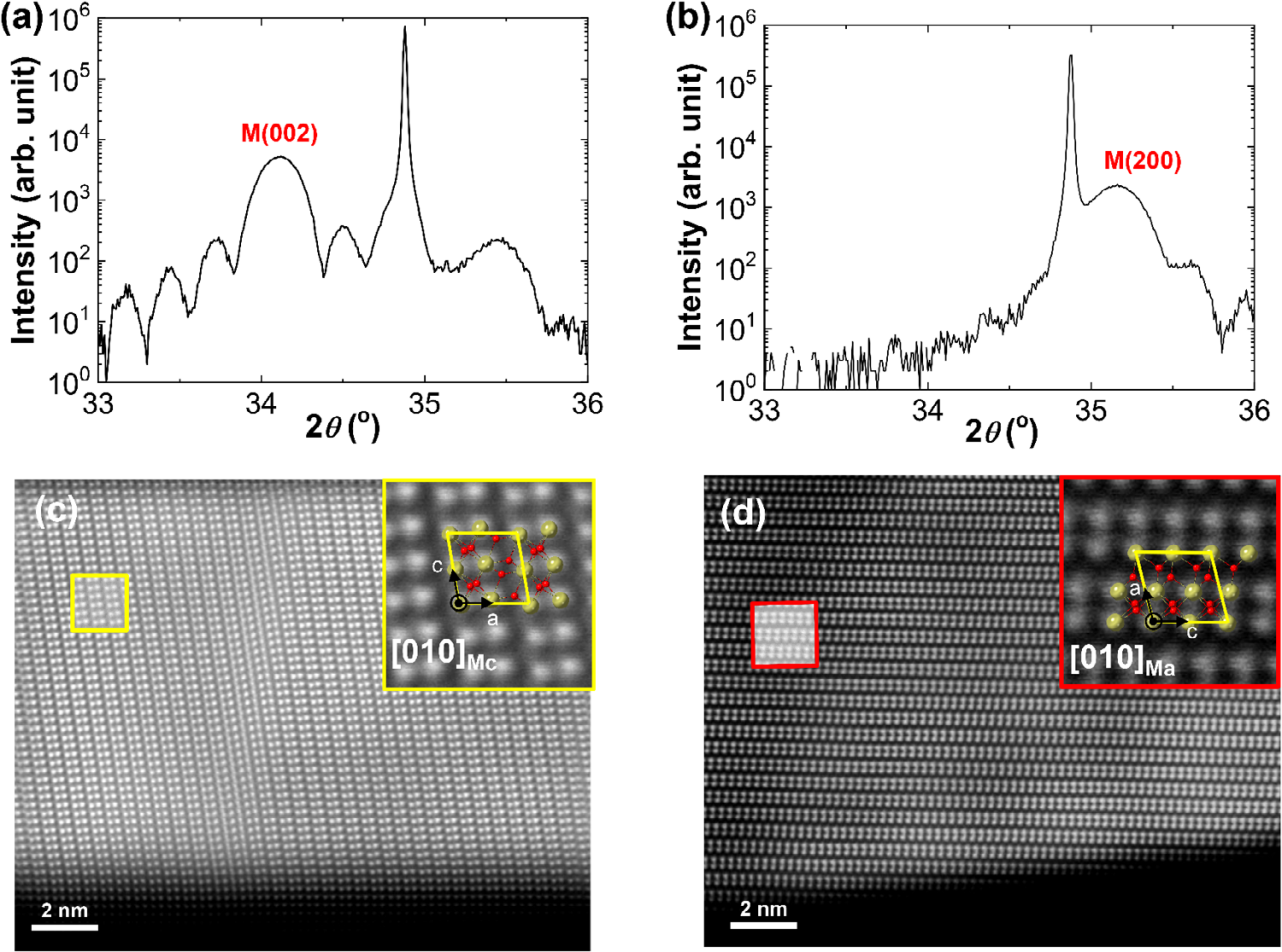
HRXRD *θ*–2*θ* scans of 30 nm‐thick epitaxial YHO thin films grown on (a) nominally‐flat and (b) 8°‐miscut YSZ substrates. Inverse fast Fourier transfer (FFT) filtering HRSTEM images of epitaxial YHO thin films grown on (c) nominally‐flat and (d) 8°‐miscut YSZ substrates. Magnified views with unit cell schematics are shown in the top‐right corners.

Figure 3 compares thick (≥ 30 nm) YHO films grown on nominally‐flat and 8°‐miscut YSZ substrates. The HRXRD *θ–*2*θ* scans reveal a clear shift in the diffraction peak positions, corresponding to out‐of‐plane interplanar spacings of 5.252 Å for the film on the flat substrate and 5.099 Å for the film on the miscut substrate. The latter peak position lies very close to that of the orthorhombic (*200*) reflection identified in Figure 2, which could lead to a misinterpretation of the phase based solely on XRD analysis. However, high‐resolution TEM imaging (Figure 3c,d) reveals instead that the film adopts a monoclinic phase with a distinct orientation—(*100*)‐oriented monoclinic—different from the (*001*) orientation observed on the nominally‐flat substrate. For the film grown on the nominally‐flat YSZ substrate (Figure 3c), the dominant orientation corresponds to the (*001*) plane of the monoclinic phase (Mc). In contrast, the film deposited on the 8° miscut YSZ substrate (Figure 3d) predominantly exhibits the (*100*) orientation of the monoclinic phase (Ma).

In the monoclinic phase, the lattice mismatch with respect to the YSZ substrate (a = 4.145 Å) along the *a*, *b*, and *c* axes are +0.6%, ‐0.5%, and ‐2.7%, respectively. Consequently, the monoclinic (*001*) plane of the YHO films is best matched to the (*001*) plane of the YSZ substrates, leading to (*001*)‐oriented monoclinic YHO films on nominally‐flat YSZ. In contrast, the high step density on the 8°‐miscut substrates provides additional restriction along the out‐of‐plane direction, promoting (*100*)‐oriented monoclinic YHO films. Notably, the monoclinic *β* angles of YHO films grown on nominally‐flat and 8°‐miscut substrates are 99° and 106°, respectively, as estimated by STEM, as shown in Figure S4. This difference reflects the unit‐cell distortion required to accommodate the lattice mismatch arising from the dense steps on the miscut substrates. These observations indicate that, in the thicker‐film regime where the monoclinic phase is stable, the substrate miscut does not change the monoclinic phase itself but instead reorients the monoclinic domains through step‐induced lattice constraints. A more comprehensive mechanism by which the step edges determine the domain structure is discussed in Section 2.5 with the aid of first‐principles calculations.

### 2.4 Miscut Effect on the Domain Structure of Ultrathin Orthorhombic YHO Thin Films

**FIGURE 4:**
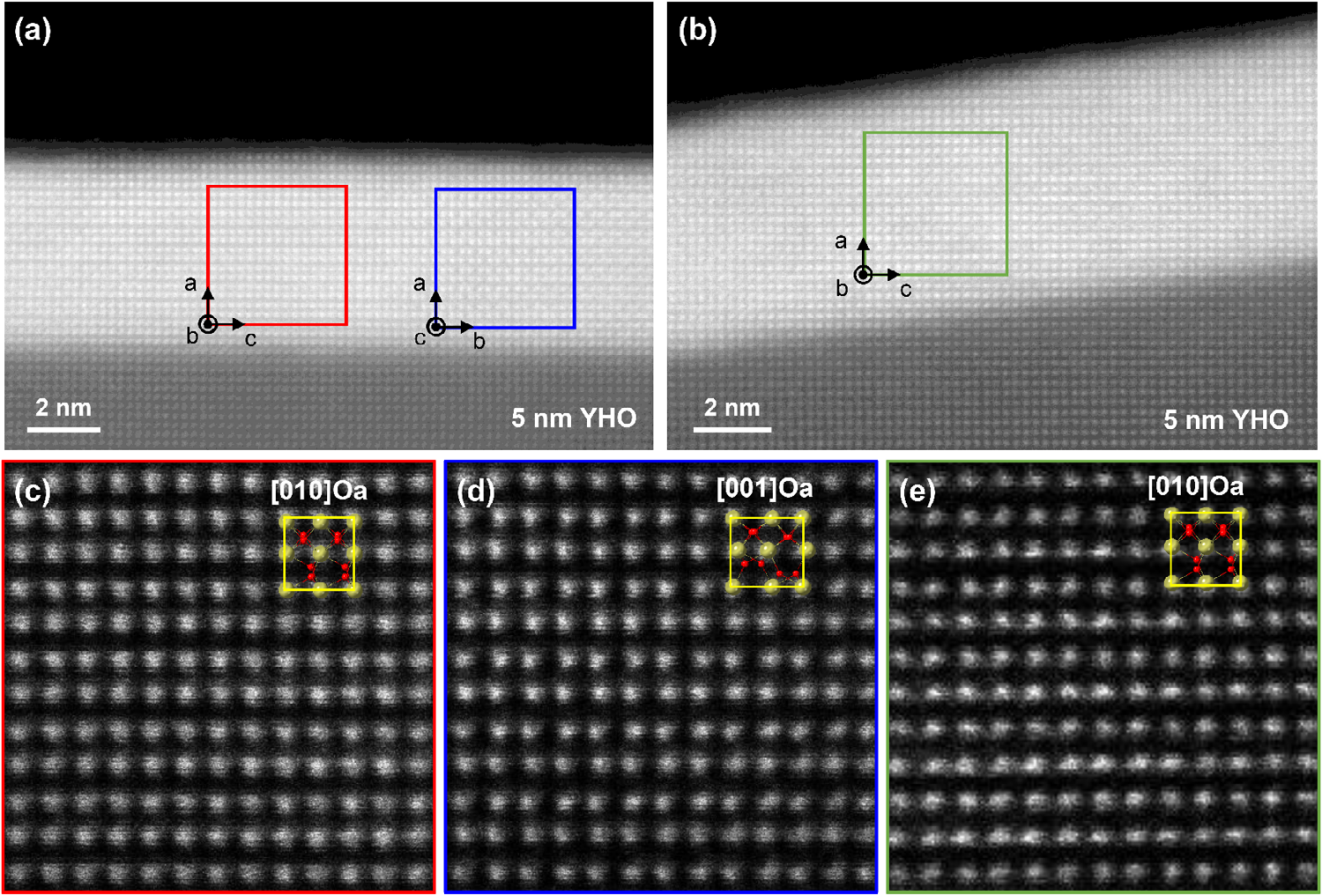
Cross‐sectional HRSTEM images of 5 nm‐thick YHO thin films grown on (a) nominally‐flat and (b) 8°‐miscut YSZ substrates. Magnified views for the regions marked in red, blue, and green in (a,b) are shown in (c–e), respectively. Schematics of the atomic arrangements are included for clarity.

Ultrathin (∼5 nm) epitaxial YHO films grown on nominally‐flat and 8°‐miscut YSZ substrates are compared in Figure 4a,b. In both cases, an orthorhombic crystal structure with a [100] out‐of‐plane orientation is stabilized; however, the domain structures depend strongly on the substrate miscut. In the orthorhombic phase, the lattice mismatch along the *a*, *b*, and *c* axes are +0.8%, ‐0.3%, and + 0.7%, respectively. Consequently, the orthorhombic (*100*) plane of the YHO films is best matched to the (*001*) plane of the YSZ substrates, leading to (*100*)‐oriented orthorhombic YHO films on nominally‐flat YSZ. Moreover, two orthorhombic domains with zone axes along [*010*] and [*001*] are observed within the TEM measurement area (Figure 4a), as indicated by [*010*]Oa and [*001*]Oa in Figure 4c,d, respectively. The coexistence of [*010*]Oa and [*001*]Oa domains on the nominally‐flat substrate reflects the intrinsic two‐fold rotational symmetry of the orthorhombic (*100*) planes, which allows energetically equivalent orientations on the cubic (*001*) surface of YSZ. In the absence of a symmetry‐breaking factor, both variants are accommodated, resulting in a two‐variant domain configuration. By contrast, the 8°‐miscut toward [100] breaks this equivalence by introducing an anisotropic step‐edge environment at the film–substrate interface. These steps serve as preferential nucleation sites, biasing the growth of [*010*]Oa domain. This preference can be attributed to the alignment of the least‐strained *b*‐axis parallel to the step edges. Thus, in the ultrathin regime, while the miscut does not modify the orthorhombic phase itself, it acts as a symmetry‐breaking mechanism that suppresses competing variants and promotes a preferential domain orientation. A more comprehensive mechanism by which the step edges determine the domain structure is discussed in Section 2.5 with the aid of first‐principles calculations.

It is worth noting that, in the ferroelectric orthorhombic phase, the spontaneous polarization is oriented along the crystallographic *c*‐axis. Because the ultrathin orthorhombic YHO films studied here adopt a (100) epitaxial orientation, with [100] out‐of‐plane on both nominally‐flat and 8°‐miscut YSZ substrates, the polar *c*‐axis necessarily lies within the film plane. Accordingly, the coexistence of [010]Oa​ and [001]Oa​ domains on the nominally‐flat substrate corresponds to two equivalent in‐plane polarization orientations. By contrast, the miscut‐induced selection of the [010]Oa​ domain fixes the in‐plane *c*‐axis along the miscut direction, indicating that substrate step geometry can serve as a practical means to deterministically control polarization orientation in epitaxial hafnia‐based ferroelectrics.

### 2.5 Experimental and Computational Study of the Miscut Effect on the Thickness–Temperature Phase Diagram

**FIGURE 5:**
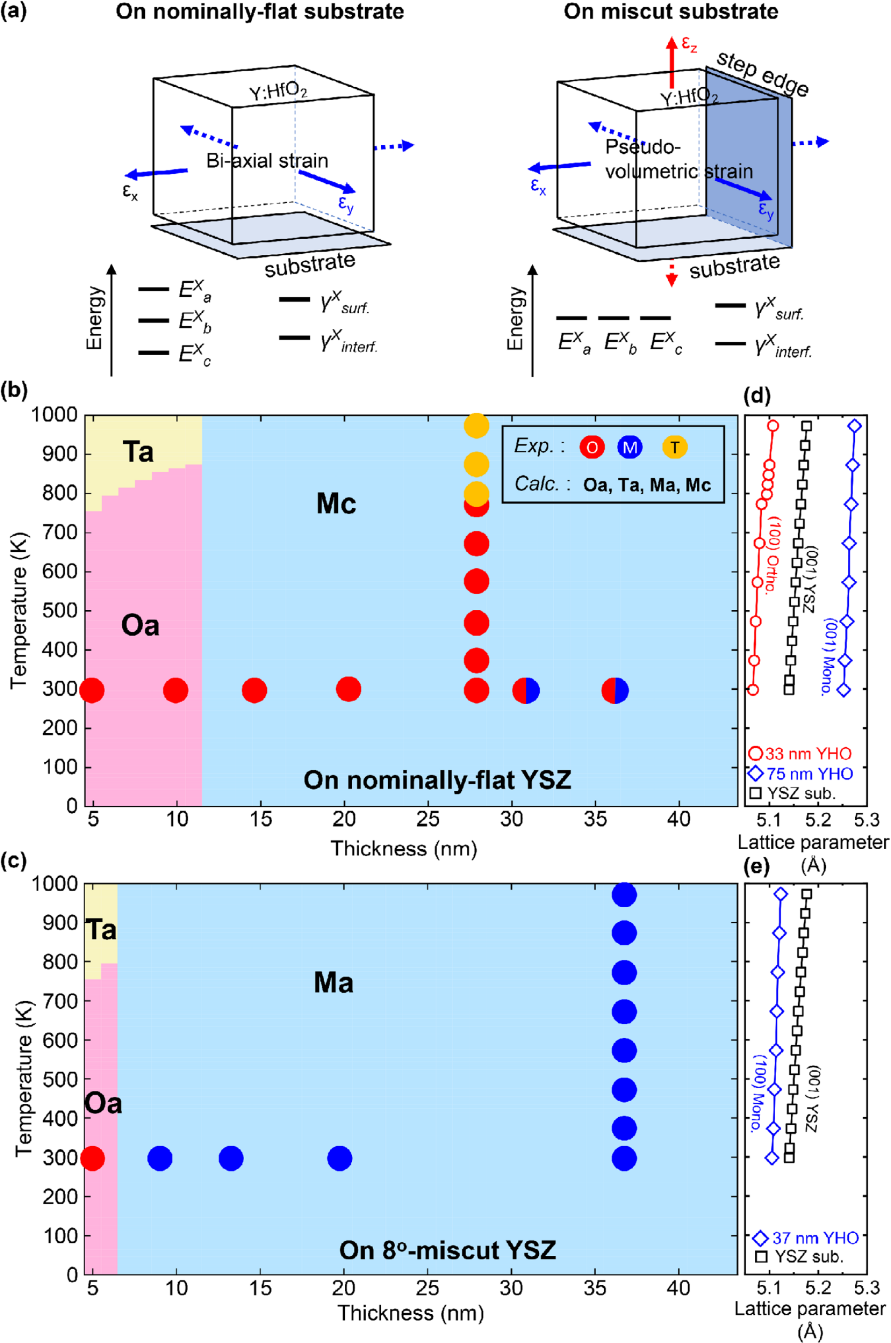
(a) Schematic illustration of the orientation‐dependent energy states of epitaxial YHO films on nominally‐flat and 8°‐miscut YSZ substrates. Here, *E^X^_a_*, *E^X^_b_* ​, and *E^X^_c_* ​ denote the bulk energies of phase X (X=monoclinic, orthorhombic, tetragonal) with the *a*‐, *b*‐, and *c*‐axes oriented along the out‐of‐plane direction, respectively. *γ^X^_surf_*_._ is the surface energy of the YHO free surface, and γ^X^_interf._​ denotes the interface energy between the YHO film and the YSZ substrate. Temperature‐thickness phase diagram of epitaxial YHO thin films grown on (b) nominally‐flat and (c) 8°‐misuct YSZ substrates. The stabilized phases obtained from the first‐principle calculations are marked by colored areas, while the experimental results are plotted as colored circles. The temperature‐dependent out‐of‐plane lattice parameters of YSZ substrates and epitaxial YHO thin films grown on the (d) nominally‐flat and (e) 8°‐misuct YSZ substrates.

Figure 5a schematically summarizes the bulk‐energy landscape under the two epitaxial boundary conditions imposed by the nominally‐flat and 8°‐miscut substrates. Here, *E^X^_a_* denotes the bulk energy of phase *X* (*X* = monoclinic, orthorhombic, or tetragonal) when the *a*‐axis is aligned along the out‐of‐plane direction; *E^X^_b_* and *E^X^_c_* are defined analogously. For a given phase‐orientation state *X*, *γ^X^_surf_* is the surface energy of the YHO free surface, and *γ^X^_interf_* ​ denotes the interface energy between the YHO film and the YSZ substrate. On the nominally‐flat YSZ substrate, the YHO film is primarily constrained by biaxial in‐plane strain. Because each phase and out‐of‐plane orientation has a different set of in‐plane lattice mismatches with the cubic YSZ surface, the strain contribution to the bulk energy differs among *E^X^_a_*, *E^X^_b_*, ​and *E^X^_c_* ​, thereby lifting their degeneracy according to the crystal symmetry, as represented by *E_bulk,2D_* in Table 1. By contrast, on the 8°‐miscut YSZ substrate, the dense step edges impose an additional lattice constraint along the out‐of‐plane direction. This step‐edge‐induced *z*‐axis strain partially transforms the biaxial strain state into a *pseudo‐*volumetric one, making the bulk energies of different orientations within a given phase nearly equivalent, as represented by *E_bulk,3D_* in Table 1. As a result, the preferred orientation on the miscut substrate is determined not only by orientation‐dependent bulk‐energy differences, but also by the combined contributions of interface and surface energies in Table 2.

**TABLE 1:** Bulk energy of Y‐doped HfO_2_. *E_bulk_*: strain‐free bulk energy, *E_bulk,2D_*: biaxially‐strained energy on nominally‐flat YSZ, *E_bulk,3D_*: volumetric strained energy on miscut YSZ.

Phase/Orientation	*E_bulk_* (eV/cell)	*E_bulk,2D_* (eV/cell)	*E_bulk,3D_* (eV/cell)
Mc	0	0	0
Ma		0.078	
Oc	0.176	0.231	0.201
Oa		0.290	
Ob		0.222	
Tc	0.402	0.496	0.441
Ta		0.447	

**TABLE 2:** Surface (γ_surf_) and interface (γ_intf_) energies of HfO_2_ strained by the YSZ lattice.

Phase /Orientation	*γ_surf_* (eV/ Å^2^)	*γ_intf_* (eV/ Å^2^)
Mc	0.119	0.059
Mb	0.149	N/A
Ma	0.130	0.021
Oc	0.200	0.040
Oa	0.117	0.021
Ob	0.144	0.007
Tc	0.109	0.009
Ta	0.125	∼0

This energetic picture provides the thermodynamic basis for the thickness–temperature phase diagrams in Figure 5b,c, which combine first‐principles calculations with experimental observations. In these calculated phase diagrams, the stable phases are indicated by color shading: light red for the orthorhombic (O) phase, light yellow for the tetragonal (T) phase, and light blue for the monoclinic (M) phase (see Experimental Section for calculation details). The corresponding stable orientations are labeled as “a,” “b,” and “c.” The calculations show that, regardless of the substrate miscut, the orthorhombic phase tends to transition to a monoclinic phase as the film thickness increases. Notably, the stability window of the orthorhombic phase becomes narrower on the miscut substrate compared to the flat substrate, suggesting that the introduction of steps promotes an earlier transition toward the monoclinic (reoriented) phase.

These features are consistent with the experimental data, which are represented by colored dots: red for the orthorhombic phase, blue for the monoclinic phase, and yellow for the tetragonal phase in Figure 5b,c. The coexistence of multiple phases is shown by mixed‐color dots composed of the corresponding phase colors. For temperature‐dependent phase evolution, high‐temperature HRXRD analyses were performed, as shown in Figure 5d,e. The 33 nm‐thick YHO film on the nominally‐flat YSZ (red plot in Figure 5d) exhibits a distinct kink in the orthorhombic (*200*) diffraction peak position at around 790 K, marking the orthorhombic to tetragonal transition. The 75 nm‐thick YHO film (blue plot in Figure 5d) shows no anomaly up to ∼1000 K, consistent with the extended stability of the monoclinic phase. The experimental results show that the orthorhombic phase is stabilized up to ∼33 nm on nominally‐flat substrates. In contrast, on the 8°‐miscut substrates, the orthorhombic phase persists only to ∼5 nm. The thicker YHO film (∼37 nm), exhibiting a (001)‐oriented monoclinic phase, shows no anomaly up to ∼1000 K (blue plot in Figure 5e), consistent with the extended stability of the monoclinic phase. Although the exact critical thickness does not coincide with the theoretical predictions, the overall correspondence between calculation and experiment is clear: both indicate that thin films preferentially stabilize the Oa phase, thicker films transform into monoclinic phases, and elevated temperatures drive the transition from Oa to the Ta phase. On the 8°‐miscut substrate, both calculation and experiment consistently reveal the appearance of the monoclinic Ma phase instead of Mc, accompanied by a narrower Oa stability window.

While ferroelectric HfO_2_ is often evaluated using metal–ferroelectric–metal (MFM) capacitors, the present work employs a bottom‐electrode‐free epitaxial platform to isolate the intrinsic role of substrate step geometry in polymorph and domain selection. Introducing a conductive underlayer (e.g., epitaxial indium tin oxide (ITO) on YSZ) is, in principle, a straightforward route toward vertical capacitor characterization; however, the large lattice mismatch and the accompanying relaxation/roughening processes, together with the distinct oxygen‐chemical‐potential requirements for Y:HfO_2_ (more oxygen‐deficient) versus ITO (more oxygen‐rich for high conductivity), make it challenging to preserve the well‐defined step–terrace morphology that is essential for a quantitative experiment–theory comparison. Because the surface step structure is the central control knob in this study, we therefore focus on structural phase/orientation evolution in the simplest boundary condition, establishing a baseline that can be extended to electrode‐integrated stacks once coherent conducting layers that retain the miscut‐defined surface morphology are realized.

Importantly, the electrode‐free configuration explored here remains highly relevant to FeFET‐type device geometries, where hafnia‐based ferroelectrics are integrated not as symmetric MFM capacitors but as part of an inherently asymmetric metal–ferroelectric–insulator–semiconductor (MFIS) gate stack. In such architectures, the semiconductor channel acts as the counter‐electrode through an interfacial dielectric, and the electrostatic boundary conditions are fundamentally different from those of an MFM capacitor. From this perspective, our results suggest a practical pathway for device‐oriented integration: epitaxial Y:HfO_2_ layer (*F*) could be grown on a crystalline YSZ buffer layer (*I*) deposited on miscut Si (*S*), allowing the miscut‐controlled step–terrace template to bias ferroelectric domain variants and polar‐axis orientation at the gate level. We expect that step‐engineering at the buffer/ferroelectric interface can therefore serve as a scalable handle to improve domain uniformity and reduce stochastic switching behavior in FeFET‐relevant stacks, providing a structural foundation for future electrical studies once conductive gate integration and processing windows are optimized.

## 3 Conclusion

We have demonstrated that substrate miscut plays a critical role in governing the phase stability, orientation, and domain structure of epitaxial 6 at.% Y‐doped HfO_2_ thin films on YSZ. By combining HRSTEM, HRXRD, and first‐principles calculations, we established thickness–temperature phase diagrams that reveal how the orthorhombic, monoclinic, and tetragonal phases vary with film thickness for both nominally‐flat and 8°‐miscut YSZ substrates. On nominally‐flat substrates, (*100*)‐oriented orthorhombic films exhibit two‐variant domain configurations and evolve into (*001*)‐oriented monoclinic phases at larger thicknesses. In contrast, the 8°‐miscut substrate stabilizes an almost single‐domain (*100*)‐oriented orthorhombic phase at small thicknesses and promotes a (*100*)‐oriented monoclinic phase at greater thicknesses, while simultaneously narrowing the thickness window in which the orthorhombic phase remains stable. These findings highlight substrate step geometry as a powerful extrinsic parameter for controlling polymorphic competition and domain structure in epitaxial HfO_2_ thin films, offering a versatile pathway for the controlled integration of diverse functional polymorphic oxides into next‐generation electronic and energy devices [36].

## 4 Experimental Section

### 4.1 Thin Film Growth

Both nominally‐flat and 8°‐miscut (001) YSZ single‐crystal substrates were used for film growth. Prior to deposition, the substrates were cleaned with acetone and deionized water, then mounted onto the substrate heater using silver paste to ensure good thermal contact. No chemical etching or thermal pre‐treatment was performed before deposition. Epitaxial YHO thin films were deposited at substrate temperatures ranging from 500°C to 750°C using a 90 ° off‐axis radio‐frequency (RF) magnetron sputtering system with 2‐inch‐diameter YHO ceramic targets containing 5, 6, or 7 at.% Y [37]. The sputtering chamber was evacuated using a turbo‐molecular pump to a base pressure of approximately 10^−6^ Torr. Deposition was conducted at a working pressure of 20 mTorr in an Ar/O_2_ gas mixture (24:0.2 sccm), with an RF power of 15 W. The growth rate of YHO films was 0.35 nm/min.

### 4.2 Characterizations

To investigate the crystal structure of YHO epitaxial thin film, HRXRD was performed using a PANalytical Empyrean diffractometer equipped with PIXcel3D multi‐channel detector. The system employed a Cu *Kα* X‐ray source (λ = 1.5406 Å) operated at 40 kV and 30 mA, with incident X‐rays monochromated using a 2‐bounce Ge (220) hybrid monochromator. *θ*–2*θ* scans were conducted to assess the out‐of‐plane crystal structure, using a step size of 0.01° and a counting time of 0.1 s per step. For temperature‐dependent HRXRD measurements, a Bruker D8 Discover diffractometer equipped with a Cu *Kα* X‐ray source (λ = 1.5406 Å) operating at 40 kV and 40 mA was used. The effective heating rate was ∼0.3°Cs^−^^1^. Each data point required approximately 5 min, consisting of ∼4 min at the target temperature (including stabilization) and ∼1 min for the *θ*–2*θ* scan. The achievable heating rate and maximum temperature were limited by the performance of the heater stage integrated into the XRD system. Surface morphology was characterized using atomic force microscopy (AFM, Digital Instruments Dimension 3100) operated in tapping mode and controlled by a Nanoscope IV controller. A thin lamella for cross‐sectional TEM analysis was prepared using a focused ion beam (FIB, Ethos NX5000, Hitachi) equipped with an Ar ion gun to remove the amorphous layer. Atomic‐scale observations—including growth direction, domain orientation, and domain boundaries—were performed using a spherical aberration‐corrected scanning transmission electron microscopy (Cs‐corrected STEM, Titan 80–300, ThermoFisher) operated at an accelerating voltage of 300 keV. The atomic arrangements were verified by comparing the observed structures with simulated HfO_2_ crystal structures.

### 4.3 Density Functional Theory

All density functional theory (DFT) calculations were performed using the Vienna Ab initio Simulation Package (VASP). The local density approximation (LDA) combined with Blöchl's projector augmented wave (PAW) method was employed to describe the electron–core interactions. A plane‐wave cutoff energy of 600 eV was applied, and the Brillouin zone was sampled using a Γ‐centered 8 × 8 × 8 k‐point mesh for the unit cell. For the doping effect of Y, a 2 × 2 × 2 HfO_2_ supercell doped with two Y atoms was employed. The most stable configuration (i.e., the lowest‐energy structure) among the possible doping arrangements was employed. The atomic positions and lattice parameters were fully relaxed until the residual Hellmann–Feynman forces were less than 0.01 eV/Å. The vibrational free‐energy was calculated using the finite‐displacement method as implemented in *Phonopy*.

Epitaxial YHO thin films were deposited on the miscut substrates, and their energy state is modeled using the equation shown in the figure:

(1) $$F_{\mathrm{grain}}=\left(E_{\mathrm{bulk}}+F_{\mathrm{vib}}\right)+\left(\gamma_{\mathrm{surf}}+\gamma_{\mathrm{intf}}\right)A_{\mathrm{xy}}$$

where *E*_bulk_ denotes the bulk energy, *F*_vib_ the vibrational Helmholtz free‐energy, *γ*_surf_ and *γ*_intf _the surface energy of the YHO film and the interfacial energy between YHO and YSZ, respectively, and *A_xy_* represents the areas of the YHO thin film in the xy plane.

Surface energies were obtained from stoichiometric and symmetric slab models, with a vacuum thickness greater than 10 Å to avoid spurious interactions between periodic images. Interface energies were evaluated using interface structures constructed with cubic ZrO_2_​, which was used to represent YSZ. For the calculation of surface and interface energies, the undoped HfO_2_ was used for simplicity [38]. The phase diagrams of Figure 5a,c were calculated using the Equation (1). The nominally‐flat and miscut YSZ substrates serve as seed layers that determine the orientation of Y:HfO_2_​, imposing biaxial and volumetric strain, respectively. On the nominally‐flat substrate, the bulk‐energy‐favored Ma​ and Ta​ phases are stabilized. In contrast, on the miscut substrate, the volumetric strain renders the bulk energies nearly equivalent, and the orientation is instead governed by minimizing the interface and surface energies, favoring the Mc​ and Ta phases. It was assumed that the YSZ substrate applies only a limited strain, affecting primarily the initial growth orientation, while the majority of the film remains essentially strain‐free. Therefore, the bulk energies used in Equation (1) correspond to strain‐free bulk energy values.

## Author Contributions

J.Y.L. and H.‐J.C. contributed equally to this work and prepared the manuscript. J.Y.L., B.L., S.O.W., and J.H.L. conducted XRD measurements. H.‐J.C., M.S.K., and D.H.H. grew epitaxial thin films. H.C. and H.J.C. performed the TEM measurements. K.H.Y. and J.‐H.C. performed the first‐principle calculations. S.H.B. conceived the idea and directed the experiments. T.H.K., H.J.C., J.‐H.C., and S.H.B. supervised the project and contributed to manuscript preparation. All authors discussed the results and contributed to the manuscript.

## Conflicts of Interest

The authors declare no conflicts of interest.

## Supporting information




**Supporting File**: advs75302‐sup‐0001‐SuppMat.docx.

## Data Availability

The data that support the findings of this study are available from the corresponding author upon reasonable request.
