# Supplementary material for: Engineering of Crystal and Domain Structures in Epitaxial Y:HfO2 Thin Films by YSZ Substrate Miscut
Source: Adv Sci (Weinh). 2026 Apr 16:e24377. Online ahead of print. doi: 10.1002/advs.202524377 (PMC13335692; doi:10.1002/advs.202524377)
Supplement: Supplementary file 1 — Supporting File: advs75302‐sup‐0001‐SuppMat.docx. [file ADVS-9999-e24377-s001.docx]

**Supporting Information**

**Engineering of Crystal and Domain Structures in Epitaxial Y:HfO_2_ Thin Films by YSZ Substrate Miscut**

Jun Young Lee^1^, Hyung-Jin Choi^1^, Kun Hee Ye^1^, Haneul Choi^2^, Byeong-hyeon Lee^3^, Min-Seok Kim^1,4^, Dong-Hun Han^1^, June Hyuk Lee^5^, Sung Ok Won^3^, Tae Heon Kim^1,6*^, Hye Jung Chang^2,6*^, Jung-Hae Choi^1*^, Seung-Hyub Baek^1,6,*^

*^1^ Electronic & Hybrid Materials Research Center, Korea Institute of Science and Technology, Seoul 02792, Republic of Korea*

*^2^ Center for Hydrogen Energy Materials, Korea Institute of Science and Technology, Seoul 02792, Republic of Korea*

*^3^ Advanced Analysis and Data Center, Korea Institute of Science and Technology, Seoul 02792, Republic of Korea*

*^4^ Department of Materials Science and Engineering, Research Institute of Advanced Materials, Seoul National University, Seoul 00826, Republic of Korea*

*^5^ Neutron Science Division, Korea Atomic Energy Research Institute, Daejeon, 34057, Republic of Korea*

*^6^ Division of Nanoscience and Technology, KIST School, Korea National University of Science and Technology, Seoul 02792, Republic of Korea*

*To whom correspondence should be addressed. E-mail: thkim79@kist.re.kr, almacore@kist.re.kr, choijh@kist.re.kr, shbaek77@kist.re.kr (S.-H. Baek)

These authors contributed equally: Jun Young Lee, Hyung-Jin Choi

**
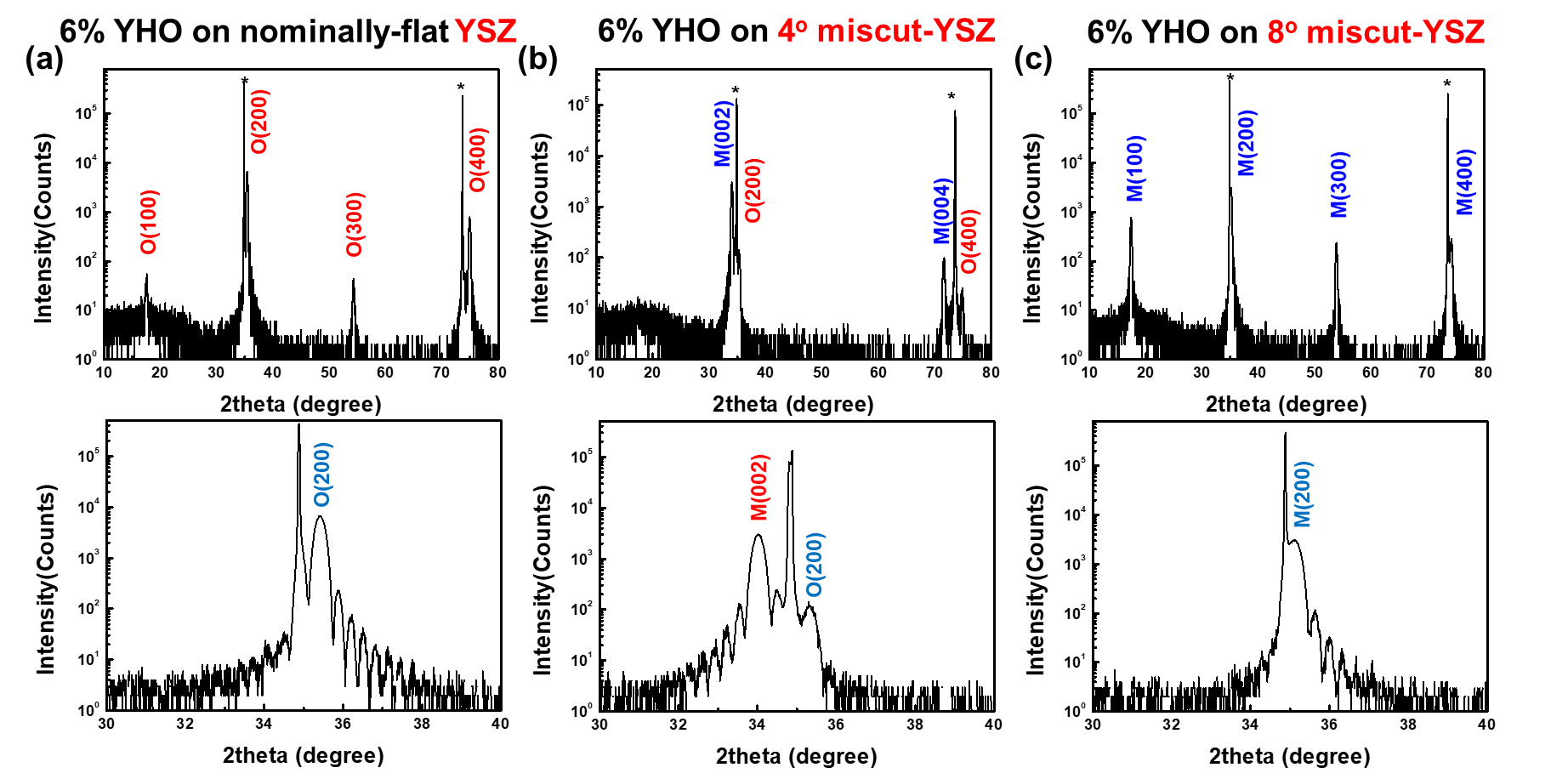
**

**Figure S1. HRXRD *θ*-2*θ* scans of epitaxial 6 at%-Y doped HfO_2_ thin films on (a) nominally-flat, (b) 4^o^-miscut, and (c) 8^o^-miscut YSZ substrates. Asterisks (*) denote diffraction peaks from the YSZ substrate.**

**
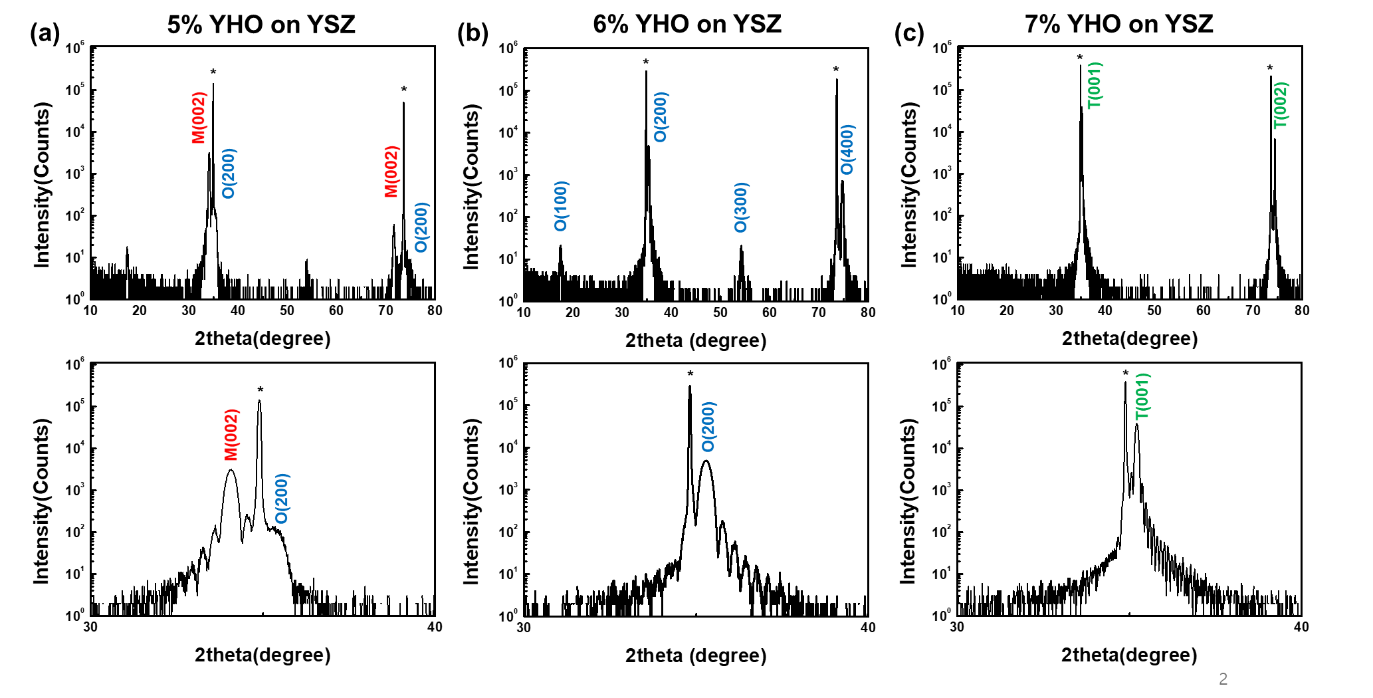
**

**Figure S2. HRXRD *θ*-2*θ* scans of epitaxial Y doped HfO_2_ thin films on nominally-flat YSZ substrates with varying Y concentrations. Panels (a)–(c) correspond to films doped with 5 at.%, 6 at.%, and 7 at.% Y, respectively. Asterisks (*) denote diffraction peaks from the YSZ substrate.**

**
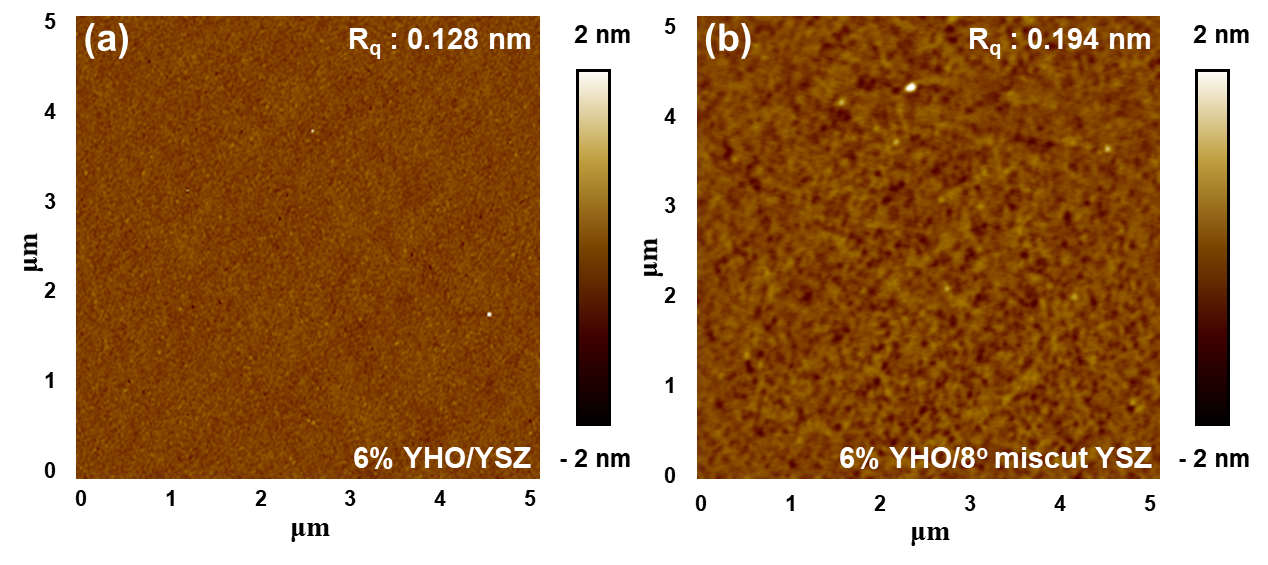
**

**Figure S3. AFM topography images of YHO films grown on (a) nominally-flat and (b) 8^o^-miscut YSZ substrates.**

**
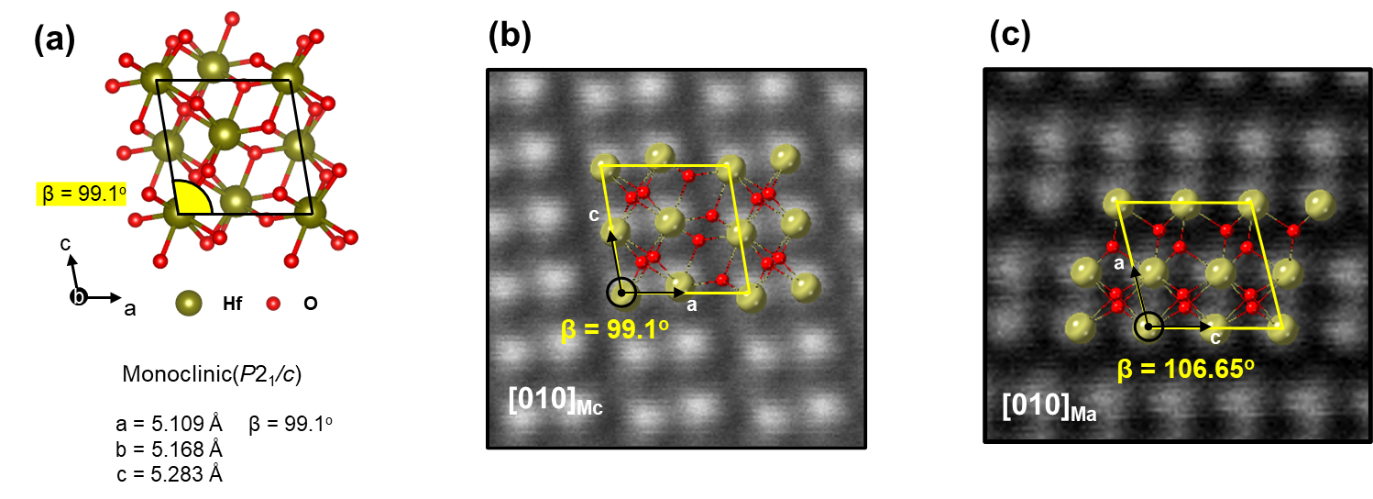
**

**Figure S4. (a) Atomic model of monoclinic YHO (*P2_1_/c*). Atomic-resolution HAADF images with superimposed monoclinic atomic models for YHO films grown on (b) a nominally flat and (c) an 8^o^-miscut YSZ substrate. A clear difference in the monoclinic b angle is observed depending on the substrate miscut, indicating a modification of the local lattice geometry.**

**
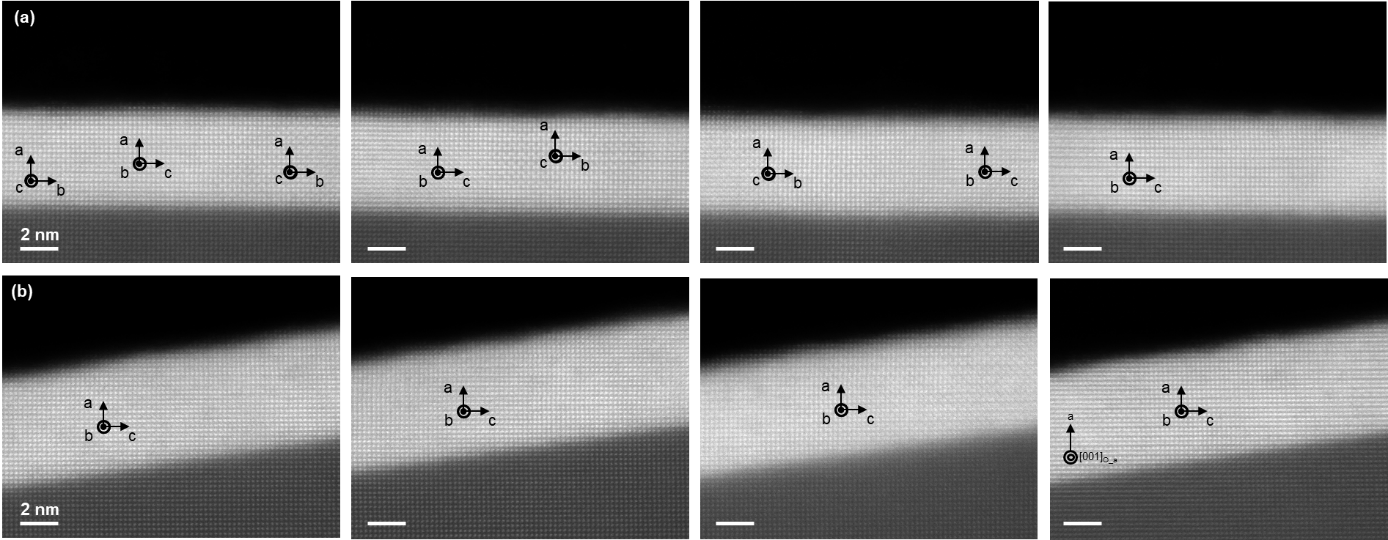
**

**Figure S5. High-magnification HAADF-STEM images collected from four different regions of 5-nm-thick epitaxial YHO thin films grown on (a) nominally-flat and (b) 8°-miscut YSZ substrates.**
